# Characterization of two Runx1-dependent nociceptor differentiation programs necessary for inflammatory versus neuropathic pain

**DOI:** 10.1186/1744-8069-6-45

**Published:** 2010-07-30

**Authors:** Omar Abdel Samad, Yang Liu, Fu-Chia Yang, Ina Kramer, Silvia Arber, Qiufu Ma

**Affiliations:** 1 Dana-Farber Cancer Institute and Department of Neurobiology, Harvard Medical School, 1 Jimmy Fund Way, Boston, MA 02115, USA; 2 Biozentrum, Department of Cell Biology, University of Basel, Klingelbergstrasse 70, 4056 Basel, Switzerland and Friedrich Miescher Institute, Maulbeerstrasse 66, 4058 Basel, Switzerland

## Abstract

**Background:**

The cellular and molecular programs that control specific types of pain are poorly understood. We reported previously that the runt domain transcription factor Runx1 is initially expressed in most nociceptors and controls sensory neuron phenotypes necessary for inflammatory and neuropathic pain.

**Results:**

Here we show that expression of Runx1-dependent ion channels and receptors is distributed into two nociceptor populations that are distinguished by persistent or transient Runx1 expression. Conditional mutation of Runx1 at perinatal stages leads to preferential impairment of Runx1-persistent nociceptors and a selective defect in inflammatory pain. Conversely, constitutive Runx1 expression in Runx1-transient nociceptors leads to an impairment of Runx1-transient nociceptors and a selective deficit in neuropathic pain. Notably, the subdivision of Runx1-persistent and Runx1-transient nociceptors does not follow the classical nociceptor subdivision into IB4^+ ^nonpeptidergic and IB4^- ^peptidergic populations.

**Conclusion:**

Altogether, we have uncovered two distinct Runx1-dependent nociceptor differentiation programs that are permissive for inflammatory versus neuropathic pain. These studies lend support to a transcription factor-based distinction of neuronal classes necessary for inflammatory versus neuropathic pain.

## Background

The sense of pain is initiated through the detection and transduction of noxious stimuli by specialized sensory neurons (called nociceptors), which are located in dorsal root ganglia (DRG) and trigeminal ganglia [1-3]. Under pathological conditions, such as tissue inflammation and nerve injury, nociceptors and central relay neurons can be sensitized by multiple pathways, leading to long-lasting chronic pain states such as inflammatory pain and neuropathic pain [2,4-8]. In the past decades, significant progress has been made in understanding the molecular and cellular bases of acute and chronic pain [9-13]. However, much less is known about how nociceptor features associated with different pain behaviors are established during development.

We and others have previously shown that Runx1, a Runt-domain transcription factor, plays a pivotal role in controlling nociceptor phenotypes and pain behaviors [14-17]. Runx1 is initially expressed in most, if not all, embryonic nociceptors marked by the expression of the neurotrophin receptor TrkA. During perinatal and postnatal development, Runx1 expression is retained in only a subset of nociceptors that switch off TrkA and turn on Ret, the receptor for the glial-derived growth factor family of neurotrophins. The remaining nociceptors switch off Runx1, most of which retain TrkA, although a subset of them expresses Ret [14]. Analyses of conditional knockout mice in which Runx1 was removed in sensory precursors show that Runx1 is required for proper development of Ret-expressing nociceptors, including the expression of dozens of sensory channels and receptors that are essential for thermal pain, inflammatory pain, and neuropathic pain [14].

A key unsolved question is which Runx1-dependent differentiation programs control which individual pain behaviors. In this study, we first made a conditional *Runx1 *knockout at perinatal stages (around embryonic day 17 or E17). In these late knockout mice, the expression of a subset of Runx1-dependent genes was affected, namely those in Runx1-persistent nociceptors, whereas expression of a separate set of Runx1-dependent genes that are normally expressed in Runx1-transient nociceptors was largely unaffected. Interestingly, behavioral analyses showed that preferential defect in Runx1-persistent nociceptors in these late knockouts led to a selective impairment of inflammatory pain without affecting neuropathic pain. Second, we made conditional knock-in mice that drive constitutive Runx1 expression in most of the nociceptors. This manipulation led to the impairment of Runx1-transient nociceptors and a selective deficit in neuropathic pain. We thus uncover two distinct Runx1-dependent differentiation programs that contribute to inflammatory versus neuropathic pain. Together, our study provides new insight into the molecular and cellular bases of chronic pain.

## Results

### Two Runx1-dependent differentiation programs revealed by analyzing late *Runx1 *conditional knockout mice: *Runx1^F/F^;Nav1.8^Cre^*

**Figure 1:**
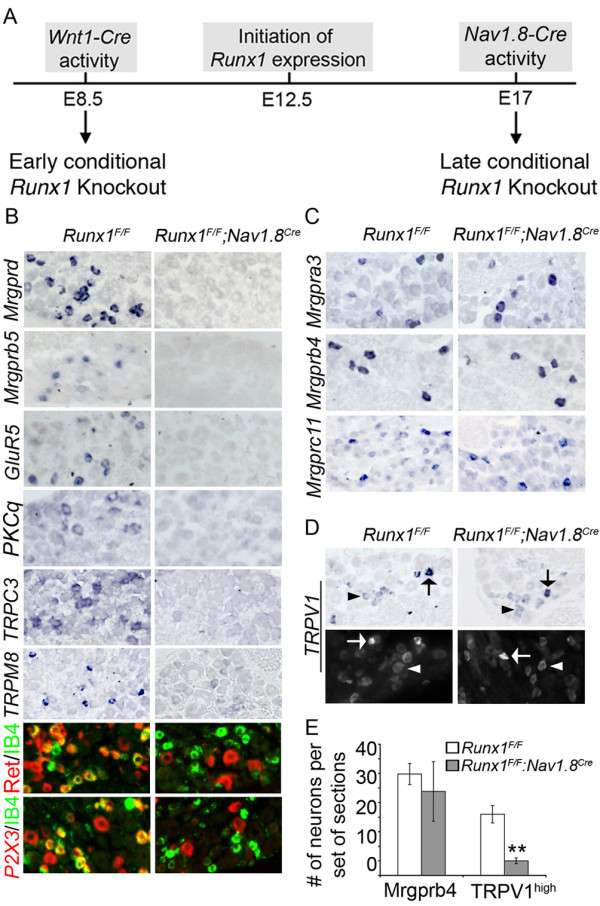
**Loss of a subset of Runx1-dependent genes in *Runx1^F/F^;Nav1.8^Cre ^*L-CKO mice**. (A) Schematic showing the timing of "early" *Runx1 *knockout (using *Wnt1-Cre *mice, removing *Runx1 *before the onset of Runx1 expression), and late *Runx1 *knockout (using *Nav1.8-Cre*, removing *Runx1 *during E16-E17). (B-D) In situ hybridization (ISH) using the indicated probes for Runx1-dependent channels and receptors on transverse sections through adult lumbar (L4/L5) DRG from *Runx1^F/F ^*control mice and *Runx1^F/F^;Nav1.8^Cre ^*L-CKO mice. For Ret and P2X3, double labeling of mRNA (red) with IB4 (green) is shown. For TRPV1 (D), both ISH (top pannels) and immunohistochemistry (IHC) (bottom panels) data are shown. The average numbers of Ret^+ ^and P2X3^+ ^neurons were decreased by 67%, from 599 ± 35 to 199 ± 10 (p < 0.01), and by 56%, from 630 ± 24 to 276 ± 30 (p < 0.001), respectively. Note that the remaining Ret^+ ^and P2X3^+ ^neurons in mutant mice are IB4^-^. (E) Graph showing the average (± SEM) of the total number of neurons expressing the indicated probes per set of lumbar DRG sections of control (white bar) and mutant (grey bar) mice. Note that the average number of Mrgprb4^+ ^neurons was not significantly changed: from 35 ± 4 to 29 ± 10 (p > 0.05). The number of TRPV1^high ^neurons (arrows) was reduced by 75%, from 21 ± 3 to 5 ± 1 (p < 0.01), whereas the number of TRPV1^low ^(arrowheads) was unchanged, from 210 ± 22 to 218 ± 10 (p > 0.05).

To determine which Runx1-dependent differentiation programs control specific types of pain, we wanted to generate mice that contain a partial impairment of Runx1-dependent nociceptor phenotypes. We made an assumption that a conditional knockout of *Runx1 *at a late developmental stage may preferentially eliminate the expression of those genes that are expressed in Runx1-persistent nociceptors. To do this, we crossed mice carrying a conditional *Runx1 *allele [18] (referred to as *Runx1^F^*) with a *Nav1.8^Cre ^*mouse line that drove Cre expression from the *Nav1.8 *sodium channel promoter in most nociceptors at perinatal stages [19]. We referred to these late *Runx1 *conditional knockout mice (*Runx1^F/F^;Nav1.8^Cre^*) as L-CKO mice. *Runx1^F/F ^*littermates were referred to as control. In lumbar DRG of L-CKO mice, Runx1 expression was not affected at E14.5, detected robustly at E16, but virtually absent at E17 (See Additional file 1). In wild-type lumbar DRG, the onset of Runx1 expression occurs at E12.5 [14]. Thus, in L-CKO mice, Runx1 operates during a developmental window from E12.5 to E17. We previously used *Wnt1^Cre ^*mice [20] to make a *Runx1 *early conditional knockout (*Runx1^F/F^;Wnt1^Cre^*), which was referred here to as E-CKO mice, in which Runx1 was removed in sensory precursors before the onset of Runx1 expression [14] (Summarized in Fig. 1A). Since pain behavior was measured from the hindpaws, all nociceptor phenotype analyses were carried out in L4-L5 lumbar DRG. Total neuron number in lumbar DRG, as measured by the expression of the panneural gene *SCG10 *[21], was not changed in L-CKO mice in comparison with that in control mice (See Additional file 1), suggesting that neuronal survival is unaffected in this knockout.

We next observed that expression of a large set of Runx1-dependent genes, revealed by loss of their expression in E-CKO mice [14] (See Additional file 2), was still eliminated in L-CKO mice (Fig. 1). These genes encode two members of the Mrgpr class of G-protein coupled receptors (Mrgprd and Mrgprb5), the ionotropic glutamate receptor GluR5/Grik1 (Fig. 1B), the protein kinase C 'theta' isoform PKCq (Fig. 1B), and two transient receptor potential channels, TRPC3 and the cold receptor TRPM8 [22]. We reported previously that Runx1 is partially required for the expression of Ret and the ATP-gated channel P2X3 [14]. Here we found that high level Ret expression and P2X3 expression were completely eliminated in IB4^+ ^neurons in L-CKO mice (Fig. 1B), whereas their expression in IB4-negative neurons was largely unaffected (Fig. 1B). TRPA1 expression in lumbar DRG was expressed at levels not detectable by our in situ hybridization method. However, using real time RT-PCR, we showed that TRPA1 expression was reduced drastically in both E-CKO mice and L-CKO mice (See Additional file 2).

Additional molecular and anatomical changes occurred in IB4^+ ^nociceptors. First, Runx1 is required to suppress the expression of a set of peptidergic nociceptor markers, such as TrkA, the neuropeptide CGRP [23], and the acid-sensing channel DRASIC [14,15,17]. Expression of these markers was expanded into IB4^+ ^neurons in E-CKO mice [14], and this expansion also occured in L-CKO mice (See Additional file 3). Second, IB4^+ ^fibers innervated the superficial lamina of the dorsal spinal cord in both E-CKO and L-CKO mice, rather than the inner lamina in control mice [14] (See Additional file 4).

In contrast, development of a separate group of Runx1-dependent sensory neuron phenotypes was no longer affected in L-CKO mice. For example, expression of several Mrgpr class GPCRs (Mrgpra3, Mrgprb4, and Mrgprc11), which was eliminated in E-CKO mice [14,24], was fully "restored" in L-CKO mice (Fig. 1C). Counting of Mrgprb4^+ ^neurons in lumbar DRG showed no differences in control versus L-CKO mice (Fig. 1E). Expression of the capsaicin receptor TRPV1 is also partially "restored" in L-CKO mice. TRPV1 expression is allocated into two separate populations of DRG neurons [25]. A small subset of DRG neurons express very high levels of TRPV1 expression (TRPV1^high^), whereas a larger subset of DRG neurons express TRPV1 at low levels [25]. Analysis of E-CKO mice showed that Runx1 is required for TRPV1^high ^expression, but dispensable for TRPV1^low ^expression [14]. In L-CKO mice, we found that TRPV1^high ^expression was only reduced by 75%, meaning that there was a 25% of "restoration" in L-CKO mice (Fig. 1D-E). Thus, by comparing mutant phenotypes in E-CKO and L-CKO mice, we have uncovered two distinct Runx1-dependent nociceptor differentiation programs, A and B. Program A controls a set of nociceptor phenotypes that are impaired in both E-CKO and L-CKO mice. Program B controls a separate set of nociceptor phenotypes that are impaired in E-CKO mice, but unchanged in L-CKO mice. Some Runx1-dependent genes are controlled by both programs, as indicated by partial "restoration" of their expression in L-CKO mice, such as TRPV1^high^.

### Program A and B Runx1 activities operate preferentially in Runx1-persistent and Runx-1 transient neurons, respectively

**Figure 2:**
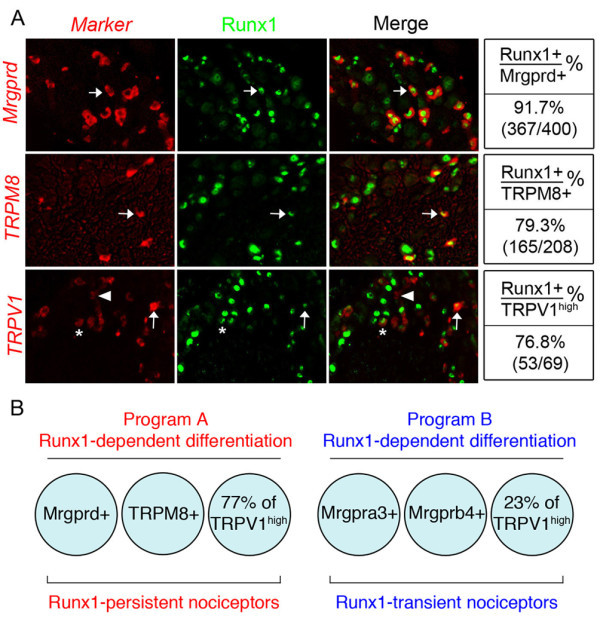
**Expression of Runx1-dependent genes in Runx1-persistent versus Runx1-transient neurons in the adult lumbar DRG**. (A) Double staining of Runx1 protein (green) and indicated RNA probe (red) on transverse sections of wild-type P30 lumbar (L5) DRG. Quantitative data are shown to the right of the panels. Note that 44.9% (231/515) and 9.2% (72/785) of Runx1^+ ^neurons coexpressed Mrgprd and TRPM8, respectively. Note also that TRPV1^high ^neurons represent 9% of total TRPV1^+ ^neurons (33/356). Arrows indicate double-labeled neurons and arrowheads indicate single-labeled neurons. For TRPV1, asterisks indicate TRPV1^low ^double-labeled neurons. (B) Schematic depicting the segregation of Runx1-dependent genes in the adult DRG into Program A and Program B, expressed predominantly in Runx1-persistent and Runx1-transient nociceptors, respectively.

To determine how program A and B Runx1 activities are related to dynamic Runx1 expression in DRG neurons, we performed a series of double staining on lumbar DRG sections, by combining Runx1 immunostaining (detecting the Runx1 protein in the nuclei) and in situ hybridization (detecting the mRNA of genes of interest in the cytoplasm). We found that a large subset of program-A dependent genes was expressed in a group of Runx1-persistent neurons marked by the expression of Mrgprd. Mrgprd^+ ^neurons represent nonpeptidergic polymodal nociceptors that respond to noxious mechanical stimuli and heat, and that innervate exclusively the skin epidermis [26,27]. We found that 92% of Mrgprd^+^neurons showed persistent Runx1 expression, and 45% of Runx1^+ ^neurons coexpressed Mrgprd (Fig. 2A). Using Mrgprd-GFP (green fluorescent protein) reporter mice, in which Mrgrpd^+ ^neurons were marked by the expression of GFP [26], we then found that expression of several other Runx1-dependent genes was confined to Mrgprd^+ ^neurons, including Mrgprb5, TRPC3, GluR5/Grik1, and PKCq (See Additional file 5). Within IB4^+ ^nociceptors, expression of P2X3 and Ret^high ^is confined to Mrgprd^+ ^neurons [26]. The selective loss of P2X3 and Ret^high ^expression in IB4^+ ^neurons in E-CKO and L-CKO mice suggests that program A-dependent P2X3 and Ret^high ^expression is also confined to Runx1-persistent Mrgprd^+ ^neurons.

The second group of program A-dependent DRG neurons is marked by the expression of TRPM8, which is involved in cold sensation [22]. Again, a majority of TRPM8^+ ^neurons (nearly 80%) showed persistent Runx1 expression, with 9% of Runx1^+ ^neurons coexpressing TRPM8 (Fig. 2A). TRPM8^+ ^neurons do not belong to the classic set of nociceptors, shown both by the little or no coexpression with CGRP and by the lack of IB4 labeling [28,29]. Thus, Runx1-persistent Mrgprd^+ ^(IB4^+^) and TRPM8^+ ^(IB4-negative) neurons represent two distinct populations of sensory neurons.

As mentioned above, TRPV1^high ^expression is controlled by both programs. We found that 77% and 23% of TRPV1^high ^neurons showed persistent and transient Runx1 expression, respectively (Fig. 2A), which matched well with 75% and 25% of TRPV1^high ^expression controlled by programs A and B, respectively. We also found that Runx1 expression was detected in 48% of TRPV1^low ^neurons (Fig. 2A), even though TRPV1^low ^expression is independent of Runx1.

Conversely, based on our previous co-localization studies [24], program B-dependent genes were expressed mainly in Runx1-transient DRG neurons. Mrgpra3^+ ^neurons have been implicated in transmitting itch evoked by chloroquine [30], and these neurons do not show persistent Runx1 expression [24]. Mrgprb4^+ ^neurons innervate exclusively the hairy skin [31], and again, these neurons do not show persistent Runx1 expression [24]. Another program B gene Mrgprc11 is expressed in both Mrgpra3^+ ^and Mrgprb4^+ ^neurons [24]. It should be noted that program B-dependent neurons can be either nonpeptidergic, such as Mrgprb4^+ ^neurons [31], or peptidergic, such as 71% of Mrgprc11^+ ^neurons (See Additional file 6). Thus in lumbar DRG, program A and program B Runx1 activities operate preferentially in Runx1-persistent and Runx1-transient DRG neurons, respectively, each of which represents a heterogeneous population of sensory neurons (Fig. 2B).

### A selective deficit of inflammatory pain in L-CKO mice

**Figure 3:**
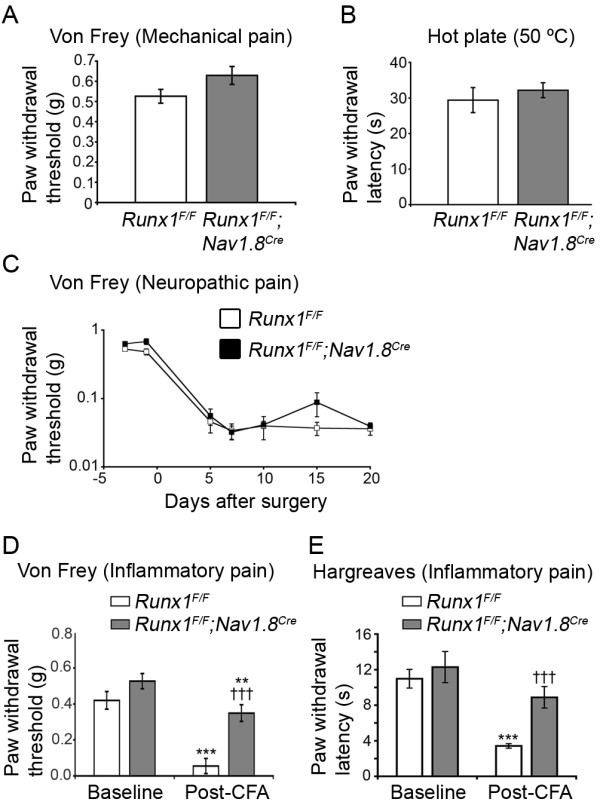
***Runx1^F/F^;Nav1.8^Cre ^*mice showed impaired inflammatory pain but largely unaffected heat pain or neuropathic pain**. (A) Mechanical thresholds measured by Von Frey filaments in control mice (n = 10) and L-CKO mice (n = 12). No difference was observed (p > 0.05). (B) Heat sensitivity measured using the hot plate assay. Controls, n = 10. Mutants, n = 12. No difference was observed (p > 0.05). (C) Neuropathic pain (SNI model). No difference was observed in mechanical allodynia over the examined time course (controls, n = 10; mutants, n = 12) (p > 0.05, ANOVA). (D, E) Inflammatory Pain (CFA model), (D) Measurement of mechanical allodynia using Von Frey filaments. Controls showed an 84 ± 3% drop in mechanical threshold two days after CFA injection (n = 9, ***p < 0.001), while mutants showed only a 33 ± 8% drop (n = 14, **p < 0.01). This difference in mechanical sensitivity drop was highly significant (^†††^p < 0.001). (E) Measurement of heat hyperalgesia using the Hargreaves apparatus. Controls (n = 8) were strongly sensitized (70 ± 4% drop in latency, ***p < 0.001) while mutants (n = 8) showed no significant sensitization (p > 0.05). The difference in latency between controls and mutants post-CFA was highly significant (^†††^p < 0.001). Error bars indicate standard errors of the mean (SEM).

We next measured responses to acute noxious heat and mechanical stimuli, as well as the sensitivity to inflammatory and neuropathic pain in L-CKO mice, with *Runx1^F/F ^*littermate mice as controls. L-CKO mice exhibited no statistically significant difference in their sensitivity to threshold mechanical stimuli (Fig. 3A), suggesting that mechanical sensitivity was unaffected in L-CKO mice as in E-CKO mice [14]. The latency in response to noxious heat (on a 50°C hot plate) was not statistically different between control mice and L-CKO mice (Fig. 3B), indicating that this type of heat pain, which was compromised in E-CKO mice (14), was retained in L-CKO mice.

We next assayed neuropathic pain induction in L-CKO mice. To that end, we used the spared nerve injury model (SNI) [32] and measured the heightened pain sensitivity in which normally innocuous tactile stimuli illicit a pain withdrawal response, a phenomenon termed mechanical allodynia that is a hallmark of neuropathic pain [8]. We found that the development of mechanical allodynia, indicated by the substantial lowering of the paw withdrawal threshold, was not significantly different between control and L-CKO mice (Fig. 3C, ANOVA, p = 0.72), indicating a nearly complete retention of this type of neuropathic pain in L-CKO mice. This result was remarkable considering a virtual abolishment of SNI-induced neuropathic pain in E-CKO mice [14].

Inflammatory pain responses were assayed by measuring the development of mechanical allodynia and heat hyperalgesia after intraplantar injection of complete Freund's adjuvant (CFA). CFA-induced edema occurred normally in both control and L-CKO mice, as shown by similar degrees of swelling in the hindpaws (see Methods). Interestingly, the development of mechanical allodynia after CFA injection was markedly impaired in L-CKO mice, shown by a substantial elevation of the paw withdrawal threshold compared with that in control littermates (Fig. 3D). We next examined how CFA-induced heat hyperalgesia was affected, using the Hargreaves assay [33]. Before CFA injection, the latency in response to radiant heat was comparable between control and L-CKO mice (Fig. 3E), consistent with the hot plate assay showing no acute heat pain deficit. However, CFA-induced heat hyperalgesia was significantly impaired in L-CKO mice, shown by a substantial increase in the latency in response to radiant heat (Fig. 3E). These results suggest that inflammatory pain is impaired in both E-CKO and L-CKO mice [14]. Thus, the selective loss of program A activity in L-CKO mice leads to a selective defect in inflammatory pain, and the retention of program B activity is sufficient to establish heat pain and neuropathic pain.

### Constitutive Runx1 expression in most nociceptors caused a selective impairment of Runx1-transient nociceptors

We next wanted to generate mice in which development of Runx1-transient nociceptors was impaired. To do this, we employed a genetic strategy that prevented Runx1 downregulation in most nociceptors. We used the conditional knock-in mice *Tau-lox-STOP-lox-Runx1-IRES-nlsLacZ-neo *[17], referred to as *Tau-Runx1^F^*. In this mouse line, Runx1 expression is under the control of the panneuronal *Tau *promoter but is not activated until the *'STOP' *cassette is removed through Cre-mediated recombination. We crossed this line with the *Nav1.8^Cre ^*transgenic mouse line [19], with resulting double heterozygous mice referred to as *Tau-Runx1^F^;Nav1.8^Cre ^*mice (See Additional file 7). In these mice, *Nav1.8^Cre ^*drove constitutive Runx1 expression in most peptidergic and nonpeptidergic nociceptors [19]. Consistently, the number of Runx1^+ ^neurons increased by 88% (See Additional file 7), implying that following constitutive Runx1 expression, a significant portion of presumably Runx1-transient sensory neurons must have survived. However, the total neuron number, marked by the expression of SCG10, was reduced by 25% (See Additional file 7**)**, suggesting some degree of neuronal cell loss. Development of proprioceptors, marked by the expression of Parvalbumin (PV), and low-threshold mechanoceptors, marked by the expression of TrkB (the receptor for the brain-derived neurotrophic factor or BDNF), was unaffected (See Additional file 8).

**Figure 4:**
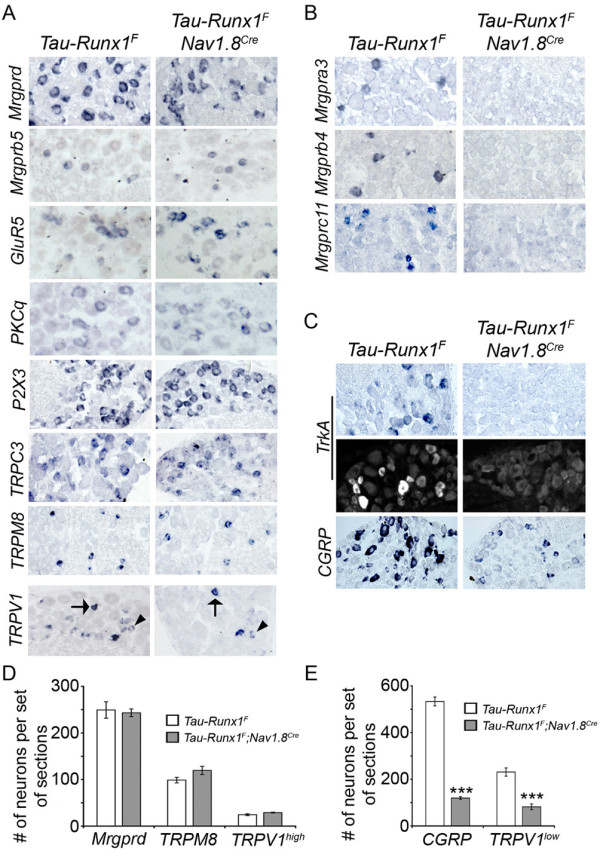
**Selective loss of Runx1-dependent genes in prospective Runx1-transient nociceptors in *Tau-Runx1^F^;Nav1.8^Cre ^*mice**. (A-C) In situ hybridization (ISH) using the indicated probes on sections through adult lumbar (L4/L5) DRG of *Tau-Runx1^F ^*control and *Tau-Runx1^F^;Nav1.8^Cre ^*mutant mice. For TrkA (C), both ISH (top pannels) and immunohistochemistry (IHC) (bottom panels) data are shown. Arrows indicate neurons expressing TRPV1^high ^and arrowheads indicate those with TRPV1^low^. (D, E) Graph showing the average (± SEM) of the total number of neurons expressing the indicated probes per set of lumbar DRG sections of control (white bar) and mutant (gray bar) mice. The numbers of Mrgprd^+^, TRPM8^+ ^and TRPV1^high ^neurons per set of sections (D) were not significantly changed in mutant versus control animals (249 ± 18 versus 243 ± 8 for Mrgprd^+ ^neurons; 99 ± 6 versus 120 ± 9 for TRPM8^+ ^neurons; and 25 ± 2 versus 29 ± 1 for TRPV1^high ^neurons) (p > 0.05). However, CGRP^+ ^and TRPV1^low ^neurons in the mutants (E) decreased from 533 ± 19 to 120 ± 6 (***p < 0.001) and from 231 ± 18 to 82 ± 12 (***p < 0.001), respectively.

We next examined nociceptor development in *Tau-Runx1^F^;Nav1.8^Cre ^*mice. Development of Runx1-persistent nociceptors was unaffected, as suggested by a normal expression of those genes predominantly expressed in Runx1-persistent nociceptors, including Mrgprd, Mrgprb5, GluR5, PKCq, P2X3, TRPC3, TRPV1^high^, and TRPM8 (Fig. 4A). Counting of Mrgprd^+ ^and TRPM8^+ ^and TRPV1^high ^neurons showed that the numbers of these neurons were comparable between *Tau-Runx1^F ^*control mice and *Tau-Runx1^F^;Nav1.8^Cre ^*mice (Fig. 4D). Furthermore, we did not observe any change in innervation of IB4^+ ^fibers to the inner lamina of the spinal cord (See Additional file 9).

Development of Runx1-transient neurons, however, was impaired in *Tau-Runx1^F^;Nav1.8^Cre ^*mice. For example, expression of a set of Runx1-dependent genes that are normally expressed in Runx1-transient neurons was eliminated, including Mrgpra3, Mrgprb4, and Mrgprc11 (Fig. 4B). The number of TRPV1^low ^neurons was reduced by 65% in *Tau-Runx1^F^;Nav1.8^Cre ^*mice (Fig. 4A and 4E), consistent with our finding that 52% of TRPV1^low ^expression is distributed in Runx1-transient neurons (Fig. 2).

TrkA^+^;CGRP^+ ^peptidergic nociceptors represent a separate group of Runx1-transient nociceptors. We had previously used *Islet1^Cre ^*to drive constitutive Runx1 expression in all DRG neurons, which resulted in a suppression of CGRP at embryonic stages without affecting embryonic TrkA expression. Those mice died at birth [17]. *Tau-Runx1^F^;Nav1.8^Cre ^*mice, on the other hand, survived into adulthood, and thus allowed us to examine postnatal development of TrkA^+^;CGRP^+ ^neurons. We found that expression of TrkA was not detected in P30 lumbar DRG of *Tau-Runx1^F^;Nav1.8^Cre ^*mice, either by in situ hybridization or by immunostaining (Fig. 4C). Expression of CGRP was also greatly reduced (by 78%) (Fig. 4C and 4E). Therefore, development of a majority of peptidergic nociceptors is impaired in *Tau-Runx1^F^;Nav1.8^Cre ^*mice. The incomplete suppression of CGRP is consistent with the observation that in wild-type DRG, about 15% of DRG neurons showed detectable Runx1 expression (See Additional file 6), suggesting that Runx1-mediated CGRP suppression operates in a cell context-dependent manner. Altogether, constitutive expression of Runx1 led to a preferential impairment of Runx1-transient nociceptors.

### A selective deficit in neuropathic pain in *Tau-Runx1^F^;Nav1.8^Cre ^*mice

**Figure 5:**
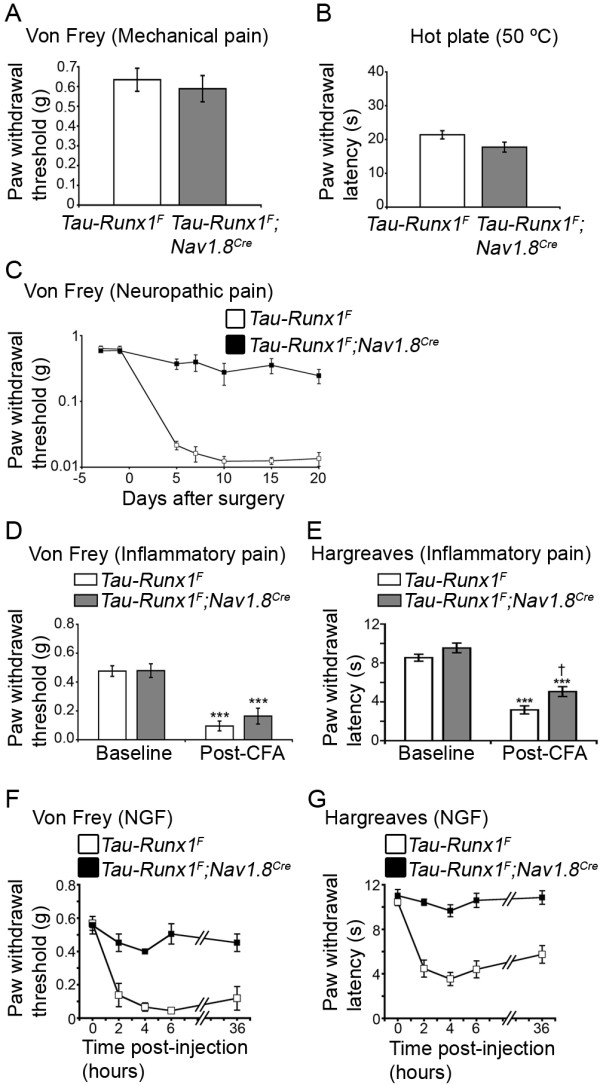
***Tau-Runx1^F^;Nav1.8^Cre ^*mice exhibited a defect in neuropathic pain but maintained normal heat and CFA-induced inflammatory pain**. (A) Mechanical thresholds measured by Von Frey filaments in controls (n = 16) and mutants (n = 10). No difference was observed (p > 0.05). (B) Heat sensitivity measured using the hot plate assay. No significant difference was observed between controls (n = 16) and mutants (n = 10) (p > 0.05). (C) Neuropathic pain (SNI). Measurement of mechanical allodynia using Von Frey filaments. A significant difference was observed over the examined time course (p < 0.001, ANOVA) between controls (n = 14) and mutants (n = 10). (D-E) Inflammatory pain (CFA), (D) Measurement of mechanical allodynia. Both controls and mutants were strongly sensitized two days after CFA injection (Controls: n = 15; ***p < 0.001. Mutants: n = 11, ***p < 0.001) with no significant difference between the two genotypes post-CFA (p > 0.05). (E) Measurement of heat hyperalgesia two days after CFA injection. Both controls and mutants were sensitized (controls: n = 5, ***p < 0.001. Mutants: n = 5; ***p < 0.001). The degree of sensitization in controls (63 ± 3% reduction in latency) was only slightly higher than that in mutants (49 ± 4%) (^†^p < 0.05). (F-G) Pain hypersensitivity in response to intraplantar NGF is impaired in mutant mice. A significant difference between controls (n = 5) and mutants (n = 4) was observed over the examined time course for both mechanical allodynia (p < 0.00001, ANOVA) (F) and thermal hyperalgesia (p < 0.00001, ANOVA) (G). Error bars indicate standard errors of the mean (SEM).

We assayed behavioral responses of *Tau-Runx1^F^;Nav1.8^Cre ^*mice to acute, neuropathic, and inflammatory pain. Mechanical sensitivity, measured by the Von Frey assay, was intact in *Tau-Runx1^F^;Nav1.8^Cre ^*mice (Fig. 5A). The mutant mice also showed no significant defect in their reaction times to 50°C noxious heat stimuli using the hot plate assay (Fig. 5B). This is consistent with the normal TRPV1^high ^expression in *Tau-Runx1^F^;Nav1.8^Cre ^*mice (Fig. 4). CFA-induced edema occurred normally, as indicated by a normal swelling in the hindpaws (see Methods). Remarkably, despite the impairment of several groups of Runx1-transient nociceptors (such as TrkA^+ ^peptidergic nociceptors), no marked deficits in CFA-induced mechanical allodynia or heat hyperalgesia were observed in *Tau-Runx1^F^;Nav1.8^Cre ^*mice (Fig. 5D and 5E). However, NGF-induced inflammatory pain was impaired (Fig. 5F and 5G). Interestingly, neuropathic pain using the SNI model was severely impaired in *Tau-Runx1^F^;Nav1.8^Cre ^*mice, as shown by a substantial increase in the paw withdrawal threshold in these mice compared to that in *Tau-Runx1^F ^*control littermates (Fig. 5C, ANOVA, p < 0.001). Thus, the impairment of Runx1-transient DRG neurons following constitutive Runx1 expression led to a selective deficit in neuropathic pain without affecting inflammatory pain (Figs. 4 and 5)

## Discussion

### Distinct modes of Runx1 activities in controlling sensory neuron development

We provide a new way to subdivide nociceptors based on persistent or transient Runx1 expression. Notably, dividing nociceptors based on Runx1 expression does not follow the classic subdivision of nociceptors, namely CGRP^+ ^peptidergic nociceptors versus IB4^+ ^non-peptidergic nociceptors [1,23]. For example, while most Runx1-persistent nociceptors, including TRPM8^+ ^and Mrgprd^+ ^neurons, are nonpeptidergic [26,28,29], about 15% of adult CGRP^+ ^neurons also show detectable Runx1 expression (See Additional file 6). Similarly, among Runx1-transient nociceptors, TrkA^+ ^and 71% of Mrgprc11^+ ^neurons are peptidergic (See Additional file 6) [23], whereas Mrgprb4^+ ^neurons are nonpeptdergic [31]. IB4 is also distributed in both Runx1-persistent and Runx1-transient nociceptors. For example, Runx1-persistent nociceptors consist of IB4^-^;TRPM8^+ ^cold receptors and IB4^+^;Mrgprd^+ ^polymodal neurons [26,28,29,34]. Similarly, Runx1-transient neurons consist of IB4^-^;TrkA^+ ^and IB4^+^; Mrgpra3/b4/c11^+ ^neurons [23,24,31].

By analyzing how Runx1-persistent and Runx1-transient nociceptors were affected in various *Runx1 *mutant mice, we recognize several distinct modes of Runx1 activity in controlling nociceptor development. The first one is observed in Runx1-persistent Mrgprd^+ ^neurons. Mrgprd expression was detected at E16 in L-CKO mice (See Additional file 2), but eliminated in adult L-CKO mice, implying that Runx1 is required to both establish and maintain these neuron identities (Fig. 1). The second one is observed in neurons expressing Mrgpra3, b4, and c11. Early Runx1 activity is necessary to establish the expression of these genes [14,24], but subsequent Runx1 extinguishment is required to maintain these neurons (Fig. 4). Mechanistically, we reported previously that Runx1 switches from an activator at early stages to a repressor at postnatal stages in regulating Mrgpra3, b4, and c11 [24], thereby explaining why their expression can only be sustained in Runx1-transient nociceptors. The third mode of action is observed in TrkA^+^;CGRP^+ ^peptidergic nociceptors. So far, it is unclear if Runx1 itself plays an essential role in these neurons. What is clear, however, is that elimination of Runx1 expression is absolutely essential for the development of TrkA^+^;CGRP^+ ^nociceptors (Fig. 4) [17]. In summary, dynamic Runx1 expression and activity play a crucial role in generating nociceptor diversity.

### Uncovering two Runx1-dependent differentiation programs necessary for inflammatory and neuropathic pain

Our studies show that Runx1-dependent nociceptor differentiation can be divided into two programs. Program A operates preferentially in Runx1-persistent nociceptors, and controls nociceptor phenotypes that are impaired in both E-CKO and L-CKO mice. Program B operates mainly in Runx1-transient nociceptors and controls a separate set of nociceptor phenotypes that are impaired in E-CKO mice but unaffected in L-CKO mice. Behavior analyses show that programs A and B are required for inflammatory pain and neuropathic pain, respectively. First, impairment of both programs in E-CKO mice led to deficits in both inflammatory and neuropathic pain [14]. Second, a selective defect in program A in L-CKO mice led to impaired inflammatory pain (CFA-induced mechanical allodynia and heat hyperalgesia), whereas the retention of program B activity in these mice is sufficient to establish neuropathic pain (SNI-induced mechanical allodynia). Third, constitutive Runx1 expression in *Tau-Runx1^F^;Nav1.8^Cre ^*mice led to impairment of Runx1-transient neurons, including program B-dependent nociceptors; these mice show a selective deficit in neuropathic pain.

Program A-dependent Runx1-persistent DRG neurons include Mrgprd^+ ^polymodal nociceptors, TRPM8^+ ^cold receptors, 75% of TRPV1^high ^neurons, and others. Mrgprd^+ ^neurons are partially required to establish mechanical hypersensitivity induced by inflammation [35]. TRPV1 is essential for inflammatory heat hyperalgesia [36,37]. Thus, the impairment of Mrgprd^+ ^neurons and the loss of 75% of TRPV1^high ^expression in L-CKO mice might contribute to the defect in CFA-induced inflammatory pain in these mice.

One surprising finding is that inflammatory pain can be established in the absence of TrkA signaling in nociceptors, despite numerous reports suggesting that TrkA signaling plays a crucial role in inflammatory pain control [4]. We found that in *Tau-Runx1^F^;Nav1.8^Cre ^*mice, despite the fact that TrkA expression was absent and NGF-induced inflammatory pain was impaired, no marked changes in CFA-induced mechanical allodynia and heat hyperalgesia were observed. Two possibilities (not necessarily mutually exclusive) are worth considering. First, TrkA signaling could be one of redundant sensitization pathways activated by CFA injection. Alternatively, TrkA-signaling may operate in immune cells to control inflammatory pain, and those cells were untouched by *Nav1.8^Cre ^*[38].

The neuropathic pain impairement observed in Runx1 gain-of-function mice (*Tau-Runx1^F^;Nav1.8^Cre ^*mice) could be due to the loss of Program B-dependent genes or be additionally caused by the impairment of other Runx1-transient neurons, such as TrkA^+^/CGRP^+ ^neurons (Fig. 4C). This complexity, however, does not affect one key conclusion of this study: Runx1-transient DRG neurons are critical for neuropathic pain, and Runx1-persistent neurons, which remain intact in Runx1 gain-of-function mice, are insufficient to allow neuropathic pain.

The neuropathic pain defect in our Runx1 gain-of-function mice seems to conflict with recent results from Abrahamsen et al., regarding the cellular basis for neuropathic pain. When using *Nav1.8^Cre ^*mice (made in the Wood lab) to ablate 85% of nociceptors, Abrahamsen et al. found that these mice showed no defect in neuropathic pain, indicating that Nav1.8-expressing neurons are dispensable for neuropathic pain [39]. However, when we used *Nav1.8^Cre ^*mice (made in the Kuner lab) to drive Runx1 expression, neuropathic pain was impaired. This discrepancy could be due to the difference between cell ablation and gene knock-in approach. Alternatively, it could be due to a specificity difference between these two Cre lines. Wood's *Nav1.8^Cre ^*mice were made by the knock-in strategy [40], whereas Kuner's *Nav1.8^Cre ^*mice were made through a transgenic approach [19]. This specificity difference is reflected by the fact that TRPM8^+ ^neurons are unaffected in Wood's ablation experiment [39], whereas TRPM8 expression was absent in *Runx1 *L-CKO mice using Kuner's *Nav1.8^Cre ^*line (Fig. 1B). Future comprehensive comparisons of these two *Cre *lines will be important for the pain field.

## Conclusion

Altogether, we have uncovered two distinct Runx1-dependent nociceptor differentiation programs. Program A operates preferentially in Runx1-persistent nociceptors and controls a set of nociceptor phenotypes necessary for inflammatory pain. Program B operates mainly in Runx1-transient nociceptors and controls a set of nociceptor phenotypes permissive for neuropathic pain. Importantly, the subdivision of Runx1-persistent versus Runx1-transient nociceptors does not follow classical subdivisions of nociceptors. Rather, our studies lend support to a transcription factor-based distinction of neuronal classes mediating inflammatory versus neuropathic pain.

## Methods

### Animals

The morning that vaginal plugs were observed was considered E0.5. For histochemical studies, adult mice at P60 (and P30 where specified) were used. For behavioral analyses, 2-3 month-old mutant and control mice were used. PCR-based genotyping was performed. Primers for the *Cre *allele and for *Runx1 *wild-type and floxed alleles have been described previously [14]. The following primers were used for the *Tau *wild-type allele, 5'-AAT GTC ACC TGC TTT AGT GGG-3' and 5'-TGG GAA GGT GAA TAT TCA ACC-3'; and for the *Neo*-containing *Tau *floxed allele, 5'-GAT CGG CCA TTG AAC AAG ATG GAT TGC-3' and 5'-AGC TCT TCA GCA ATA TCA CGG GTA GCC-3'. All animal handling, surgeries, and behavioral test protocols (described below) were approved by the Institutional Animal Care and Use Committee at Dana-Farber Cancer Institute.

### Real-time RT-PCR analysis of gene expression

Two biologically duplicated sets of total RNA were isolated from lumbar DRG of adult *Runx1^F/F^*, *Runx1^F/F^;Wnt1^Cre^*, or *Runx1^F/F^;Nav1.8^Cre ^*mice using TRIZOL (invitrogen, USA) and following the manufacturer's protocol. Reverse transcription was performed with 2.5 μg of RNA by using Superscript III first strand synthesis kit (Invitrogen, USA). Real-time quantitative PCR was then performed using SYBR green master mix (Invitrogen, USA) in a 7500 Real-time PCR machine (Applied Biosystems, USA). The following primer pairs were used: TRPA1 (Forward: 5'-GGAGACCCTGCTTCACAGAG-3', Reverse: 5'-AGTGGAGAGCGTCCTTCAGA-3'), HPRT (Hypoxanthine phosphoribosyl transferase) (Forward: 5'-GGCCAGACTTTGTTGGATTTG-3', Reverse: 5'-TGCGCTCATCTTAGGCTTTGT-3')

### In Situ Hybridization and Immunostaining

Tissue preparation, the in situ hybridization (ISH) procedure, and the probes for TRPM8, TRPV1, Mrgprc11, CGRP, Mrgprd, TRPC3, P2X3, Mrgpra3, Mrgprb4, TrkA, SCG10, DRASIC, Ret, and GluR5/Grik1, have been described previously [14,24,41]. The probes for PKCq and Mrgprb5 were amplified with gene specific sets of PCR primers from cDNA template from adult mouse DRG. Immunohistochemistry (IHC) using rabbit anti-Runx1 (T. Jessell, Columbia University), rabbit anti-TrkA (L. Reichardt, UCSF) or IB4-biotin (10 μg/ml, Sigma) was carried out as previously described [14]. IHC using rabbit anti-TRPV1 (1/4000, AbCam, USA) was performed on floating sections as described previously [42]. ISH combined with rabbit anti-Runx1, rabbit anti-CGRP (Peninsula, USA) or IB4-biotin staining has been previously described [14]. Fluorescent immunostaining images were photographed first, followed by the development of the ISH signals. The brightfield images of ISH signals were inverted and then merged with the fluorescence images. This sequential photographing avoids the masking of low-level fluorescent signals by non-fluorescent in situ signals, leading to a more sensitive detection of the coexpression of Runx1, CGRP, or IB4 with genes of interest. For GFP/ISH double stainings, GFP fluorescent images were directly photographed on sections (in RNase-free PBS solution) and then ISH was carried out as described above. The brightfield images of ISH signals were inverted, and then merged with the fluorescence images.

### Cell Counting

To count DRG neurons, L4/L5 lumbar DRG were dissected from two to three pairs of mutant and control mice, sectioned in a series of eight slides at a 12 μm thickness, and each set processed for immunostaining or used for ISH [14]. The number of neurons per set of sections was reported. Only cells containing nuclei and showing levels of expression clearly above background were counted. At least three independent L4 or L5 lumbar DRG were used for each counting. Averages and standard errors of the mean (SEM) were calculated, and the difference between wild-type and mutant samples was subjected to a Student's t test (Two-Sample Assuming Unequal Variance), with p < 0.05 considered significant.

### Surgery

The spared nerve injury (SNI) model for neuropathic pain was performed on adult mice (P60 to P90) as described for rats [32]. The animals used were *Runx1^F/F ^*control and *Runx1^F/F^;Nav1.8^Cre ^*mutant mice or *Tau-Runx1^F ^*control and *Tau-Runx^F^;Nav1.8^Cre ^*gain-of-function mutant mice. Briefly, animals were anesthetized with an IP injection of 30 μl of nembutal sodium solution (50 mg/ml, Ovation) or by exposure to isofluorane (2%). An incision was made on the lateral mid-thigh, and the underlying muscle was separated to expose the sciatic nerve. The three branches of the sciatic nerve (tibial, common peroneal, and sural nerves) were carefully separated while minimizing any contact with or stretching of the sural. The tibial and common peroneal nerves were then individually ligated with 6.0 silk sutures and cut distally. 2-3 mm distal to the ligation of each of the tibial and common peroneal nerves were removed. The muscle and skin incisions were closed with silk sutures (6.0). For CFA-mediated inflammation, mice were briefly anesthetized with isofluorane (3-5 min at 2%), and 15 μl of Complete Freund's Adjuvant (CFA) (Sigma) were injected into the plantar surface of the left hindpaw. The thickness of the feet, before and two days after CFA injection, was measured to examine edema development. In 9 *Runx1^F/F ^*control mice and 14 *Runx1^F/F^;Nav1.8^Cre ^*mutant mice, the thickness of the feet increased similarly from 2.31 ± 0.06 mm to 2.90 ± 0.09 mm (t-test, p < 0.001), and from 2.24 ± 0.09 mm to 2.99 ± 0.07 mm (t-test, p < 0.001), respectively. There was no significant difference in the thickness of CFA-injected paws between these two genotypes (t-test, p > 0.05). In 15 *Tau-Runx1^F ^*control mice and 11 *Tau-Runx1^F^;Nav1.8^Cre ^*mutant mice, the thickness of the feet increased similarly from 2.61 ± 0.02 mm to 3.24 ± 0.05 mm (t-test, p < 0.01), and from 2.36 ± 0.09 mm to 3.10 ± 0.11 mm (t-test, p < 0.001), respectively. There was no significant difference in the thickness of CFA-injected paws between these two genotypes (t-test, p > 0.05). For NGF-mediated inflammation, 10 μl of NGF (Sigma, USA) diluted in saline at 5 ng/μl was injected into the plantar surface of the left hindpaw.

### Behavior

All animals were acclimatized to the behavioral testing apparatus in three to five 'habituation' sessions. After habituation, two baseline measures were recorded on two consecutive days for each of the behavioral tests prior to the surgery. After the surgical procedures (considered as day 0), the behavioral tests were performed at defined intervals (see Figs. 3 and 5). The experimenter was blinded to the genotype of the animals. To measure mechanical pain, we placed the animals on an elevated wire grid and the lateral plantar surface of the hindpaw was stimulated with calibrated Von Frey monofilaments (0.008-2 g). We started with the 0.16 g filament and moved up if response is negative and down if response is positive. The withdrawal threshold for the Von Frey assay was determined as the filament at which the animal withdrew, flicked or licked its paw at least twice in ten applications. To measure surface heat pain, we placed mice on a hot plate (IITC, USA) and the latency to hindpaw flicking, licking, or jumping was measured. The hot plate was set at 50°C, and all animals were tested sequentially with at least 5 min between tests. A cutoff time of 60 seconds was set for testing at 50°C. To measure radiant heat pain, animals were put in plastic boxes and the plantar paw surface was exposed to a beam of radiant heat (IITC, USA) according to the Hargreaves method [33]. Paw withdrawal latency was then recorded (beam intensity was adjusted to result in a latency of 8-12 seconds for control animals baselines). The heat stimulation was repeated 3 times at an interval of 5-10 min for each animal and the mean calculated. A cutoff time of 18 seconds was set to prevent tissue damage.

### Statistical Analyses

Baseline data were calculated as the average of two independent tests performed on two consecutive days. Post-CFA data were taken from a single test performed two days post-injection and subjected to the Student's t-test (Two-Sample Assuming Unequal Variance). Post-SNI Von Frey time course measurements for *Runx1^F/F ^*compared to *Runx1^F/F^;Nav1.8^Cre ^*and *Tau-Runx1^F ^*compared to *Tau-Runx1^F^;SNS^Cre ^*were analyzed by two-way repeated ANOVA (R, R Development Core Team, Austria) followed by Bonferroni's posttest. Von Frey results in SNI animals were plotted using a log scale. p < 0.05 was accepted as statistically significant. Post-NGF time course measurements (Von Frey and Hargreaves) for *Tau-Runx1^F ^*compared to *Tau-Runx1^F^;SNS^Cre ^*were analyzed by two-way repeated ANOVA (R, R Development Core Team, Austria) followed by Bonferroni's posttest.

## Competing interests

The authors declare that they have no competing interests.

## Authors' contributions

QM conceived and supervised the conduct of the study. OAS, YL, and FCY carried out all the molecular analyses. OAS, with contribution from YL and FCY, performed all behavioral experiments. OAS, YL, FCY, and QM analyzed the molecular and behavioral data. IK and SA contributed reagents. OAS and QM drafted the article, with help in editing from FCY. All authors read and approved the final manuscript.

## Supplementary Material

Additional file 1 **Generation of *Runx1^F/F^;Nav1.8^Cre ^*late conditional knockout (L-CKO) in the DRG**. (A) Schematic showing the conditional *Runx1 *allele. Exon 4, encoding part of the DNA binding Runt domain, was flanked by two loxP sequences (black triangles). Deletion of this exon generated a null allele. The neo cassette was also flanked by loxP sites. After crossing *Runx1^F/F ^*mice with *Nav1.8^Cre ^*mice, exon 4 and the neo cassette were excised by Cre-mediated DNA recombination. This *Runx1 *conditional knockout mouse line was referred to as *Runx1^F/F^;Nav1.8^Cre^*. (B) Runx1 expression was unchanged in E14.5 L-CKO mutant mice. Double immunostaining of Runx1 (green) and TrkA (red) at E14.5 on lumbar DRG sections from control mice and mutant mice (left). Note that at this stage the percentage of Runx1^+ ^in TrkA^+ ^neurons was unchanged, from 91% (631/687) in control mice to 92% (434/471) in mutant mice as shown in the graph (right). (C) Runx1 expression was eliminated by E17. Immunostaining of Runx1 on sections through lumbar L-CKO DRG at E16 (left) and E17 (right). Note that Runx1 expression was still strongly detected at E16 but completely lost at E17. (D) L4-L5 DRG total neuronal number was not changed in *Runx1 *late knockouts. Graph showing that the total number of neurons (as determined by the expression of the panneuronal marker SCG10) per set of sections in P30 lumbar DRG in control versus mutant mice was not significantly changed (from 1285 ± 50 versus 1314 ± 40 respectively, p > 0.05). Click here for file

Additional file 2 **Runx1 was required for Mrgprb5, GluR5, PKCq, and TRPA1 expression, and for the maintenance of Mrgprd expression**. (A) In situ hybridization using the indicated probe on lumbar L4/5 DRG from P30 *Runx1^F/F ^*control mice and *Runx1^F/F^;Wnt^Cre ^*early knockout mice. (B) Graph shows RT-PCR measuring mRNA levels of TRPA1 using adult L4-5 DRG from *Runx1^F/F ^*control, *Runx1^F/F^;Wnt1^Cre ^*early KO, and *Runx1^F/F^;Nav1.8^Cre ^*late KO mice. RT-PCR data was normalized with HPRT. Each bar represent n = 2 mice. (C) In situ hybridization using an Mrgprd probe on sections through E16.5 lumbar DRG of *Runx1^F/F ^*control and *Runx1^F/F^;Nav1.8^Cre ^*mutant mice. Note that Mrgprd was detected in the mutant at E16.5 but lost at P30 (see Fig. 1), indicating a maintenance role. Click here for file

Additional file 3 **Expression of Ret, TrkA, CGRP and DRASIC in *Runx1^F/F^;Nav1.8^Cre ^*late knockout mice**. (A) In situ hybridization with a Ret probe on transverse sections through adult lumbar DRG of control *Runx1^F/F ^*mice and mutant *Runx1^F/F^;Nav1.8^Cre ^*mice. To the right of the panels, a graph showed that the average (± SEM) number of neurons expressing Ret per set of lumbar DRG sections was reduced from 630 ± 24 (control, white bar) to 276 ± 30 (mutant, gray bar) (p < 0.001). (B) Expansion of peptidergic neuron markers in late *Runx1 *knockout. Single In situ hybridization with indicated probes (top) or double labeling of indicated mRNA (red) with IB4 (green) (bottom) in control and mutant DRG. The number of TrkA^+ ^neurons increased from 262 ± 18 to 900 ± 46 (p < 0.001), and TrkA expression in IB4^+ ^neurons increased from 5.30% ± 0.05% to 80.4% ± 2.4% (p < 0.05). CGRP^+ ^neurons increased from 434 ± 23 to 1171 ± 52 (p < 0.001), and CGRP^+ ^in IB4^+ ^neurons increased from 11.4% ± 0.4% to 81.5% ± 1.3% (p < 0.01). DRASIC^+ ^in IB4^+ ^neurons increased from 0% to 57.1% ± 4.4% (p < 0.001). However, the expression of the vesicular Glutamate transporters (VGLUT1 and VGLUT2) and Nav1.8 were not affected in late *Runx1 *knockout mice (data not shown). Click here for file

Additional file 4 **Afferent central innervation of IB4^+ ^sensory fibers in the dorsal horn was impaired in *Runx1^F/F^;Nav1.8^Cre ^*late knockout**. Double staining of IB4 (green) and CGRP (red) on P30 dorsal horn sections of control *Runx1^F/F ^*and *Runx1^F/F^;Nav1.8^Cre ^*mice. In control mice, peptidergic CGRP^+ ^fibers innervated the superficial lamina, while nonpeptidergic IB4^+ ^fibers innervated preferentially the inner lamina. Similar to the phenotype in *Runx1 *early conditional mutant, IB4^+ ^fibers in *Runx1^F/F^;Nav1.8^Cre ^*late mutants shifted their innervation from the inner lamina to the more superficial lamina. Click here for file

Additional file 5 **Double labeling of GFP protein (green) and indicated RNA probe (red) in lumbar DRG from P30 *Mrgprd^ΔEGFP ^*animals**. Mrgprd was used here as a surrogate marker for Runx1^+ ^neurons; see main text for details. Note that Mrgprb5, GluR5, TRPC3, and PKCq were largely overlapping with GFP and were thus predominantly in Runx1-persistent neurons. Quantitative data were shown to the right of the panels. Click here for file

Additional file 6 **Double Staining of CGRP with Mrgprc11 and with Runx1**. (A) A majority of Mrgprc11^+ ^neurons were peptidergic. Double staining of CGRP protein (red) and Mrgprc11 mRNA (green) on sections from P30 lumbar DRG from WT mice. Note that 71% of total Mrgprc11^+ ^neurons (67 of 94) coexpressed CGRP (arrows). (B) Runx1 was expressed in a small subset of peptidergic neurons. Double labeling of Runx1 protein (green) and CGRP mRNA (red) on P30 lumbar sections of DRG from WT mice. About 15% of CGRP^+ ^neurons (40 in 261) showed detectable Runx1 expression (arrows). Click here for file

Additional file 7 **Generation of *Tau-Runx1^F^;Nav1.8^Cre ^*mice that drove constitutive Runx1 expression in most nociceptors**. (A) Schematic showing the conditional knock-in of the *Tau-Runx1 *allele. A *lox-STOP-lox-Runx1-IRES-nlsLacZ-neo *cassette was inserted into exon 2 (black box) of the *Tau *locus. After crossing *Tau-Runx1^F ^*mice with *Nav1.8^Cre ^*mice, The 'STOP' was excised by Cre-mediated DNA recombination, allowing Runx1 to be expressed from the *Tau *locus. (B) Expansion of Runx1 expression in *Tau-Runx1^F^;Nav1.8^Cre ^*mutant mice. Transverse sections through adult lumbar DRG of *Tau-Runx1^F ^*control mice (left) and *Tau-Runx1^F^;Nav1.8^Cre ^*mice (right) were labeled by immunostaining with Runx1. Arrowheads indicate Runx1-negative neurons while arrows indicate Runx1-positive neurons. (C) Graph showing that the average (± SEM) of total number of neurons expressing Runx1 (as detected by immunohistochemistry) per set of sections from control (white bar) and mutant mice (gray bar) increased from 460 ± 14 to 864 ± 17 (***p < 0.001). Futhermore, the total neuron number (as marked by the pan-neuronal marker SCG10) was reduced in the mutant mice by 25% (from 1427 ± 52.8 to 1068 ± 33, p < 0.01). Click here for file

Additional file 8 **Development of proprioceptors and mechanoceptors was unaffected in *Tau-Runx1^F^;Nav1.8^Cre ^*mice**. (A) In situ hybridization using the indicated probes on sections through adult lumbar (L4/L5) DRG of *Tau-Runx1^F ^*control and *Tau-Runx1^F^;Nav1.8^Cre ^*mutant mice. (B) Graph showing the average (± SEM) of the total number of neurons expressing the indicated probes per set of lumbar DRG sections of control (white bar) and mutant mice (gray bar). Note that the numbers of PV^+ ^and TrkB^+ ^neurons per set of sections were not significantly changed in mutant versus control animals (from 177 ± 11 to 175 ± 5 for PV^+ ^neurons and from 48 ± 2 to 42 ± 4 for TrkB^+ ^neurons) (p > 0.05). Click here for file

Additional file 9 **Examination of afferent central innervation in the dorsal horn of *Tau-Runx1^F^;Nav1.8^Cre ^*mutants**. Double staining of IB4 (green) and CGRP (red) on P30 dorsal horn sections of *Tau-Runx1^F ^*control and *Tau-Runx1^F^;Nav1.8^Cre ^*mutant mice. Note that the residual CGRP^+ ^fibers still innervated the superficial lamina, and IB4^+ ^fibers also showed normal innervation to the inner lamina of the spinal cord of mutant mice. Click here for file
