# Supplementary figures and images for: Characterization of two Runx1-dependent nociceptor differentiation programs necessary for inflammatory versus neuropathic pain
Source: Mol Pain. 2010 Jul 30;6:45. doi: 10.1186/1744-8069-6-45 (PMC2919460; doi:10.1186/1744-8069-6-45)

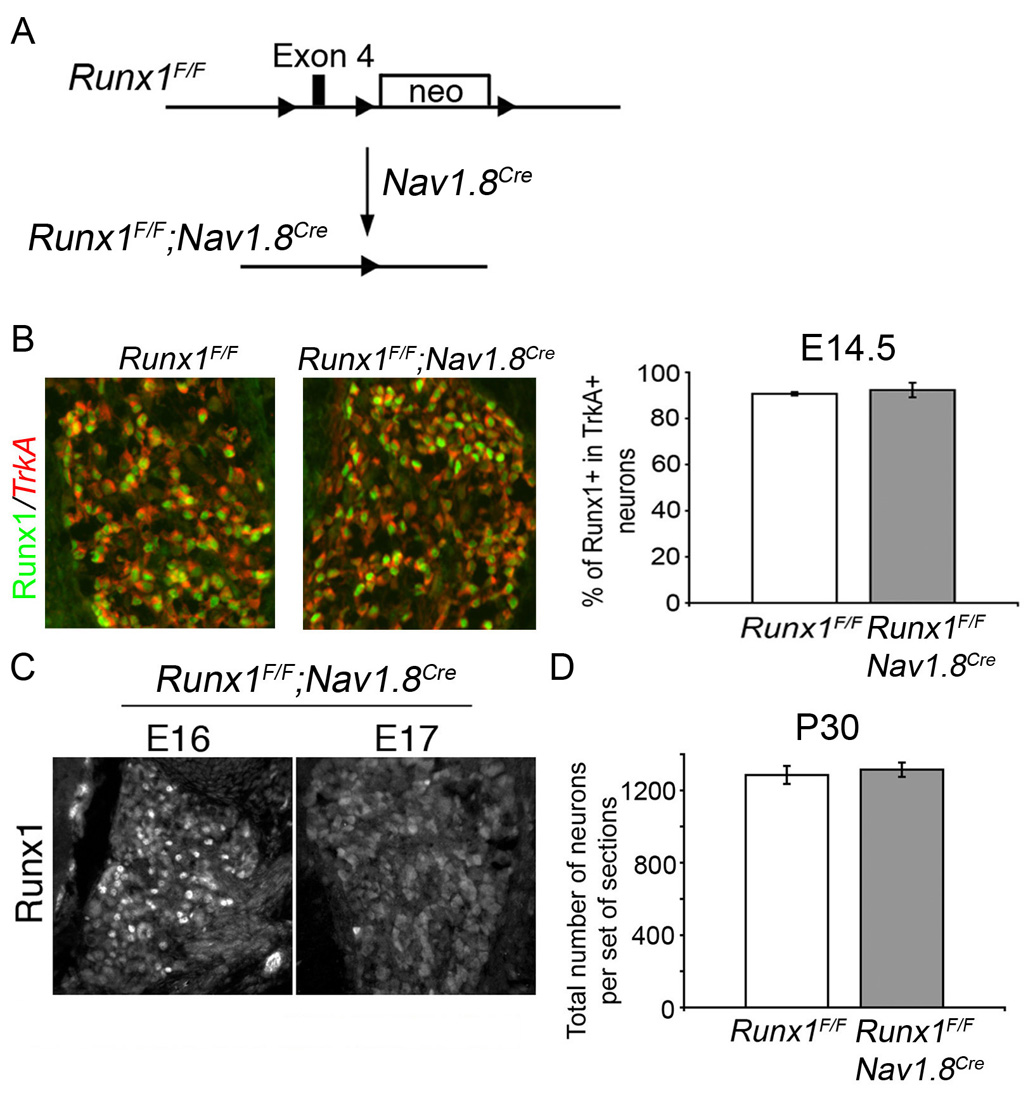

Supplement: Additional file 1 — Generation of Runx1F/F;Nav1.8Cre late conditional knockout (L-CKO) in the DRG. (A) Schematic showing the conditional Runx1 allele. Exon 4, encoding part of the DNA binding Runt domain, was flanked by two loxP sequences (black triangles). Deletion of this exon generated a null allele. The neo cassette was also flanked by loxP sites. After crossing Runx1F/F mice with Nav1.8Cre mice, exon 4 and the neo cassette were excised by Cre-mediated DNA recombination. This Runx1 conditional knockout mouse line was referred to as Runx1F/F;Nav1.8Cre. (B) Runx1 expression was unchanged in E14.5 L-CKO mutant mice. Double immunostaining of Runx1 (green) and TrkA (red) at E14.5 on lumbar DRG sections from control mice and mutant mice (left). Note that at this stage the percentage of Runx1+ in TrkA+ neurons was unchanged, from 91% (631/687) in control mice to 92% (434/471) in mutant mice as shown in the graph (right). (C) Runx1 expression was eliminated by E17. Immunostaining of Runx1 on sections through lumbar L-CKO DRG at E16 (left) and E17 (right). Note that Runx1 expression was still strongly detected at E16 but completely lost at E17. (D) L4-L5 DRG total neuronal number was not changed in Runx1 late knockouts. Graph showing that the total number of neurons (as determined by the expression of the panneuronal marker SCG10) per set of sections in P30 lumbar DRG in control versus mutant mice was not significantly changed (from 1285 ± 50 versus 1314 ± 40 respectively, p > 0.05). [file 1744-8069-6-45-S1.TIFF]

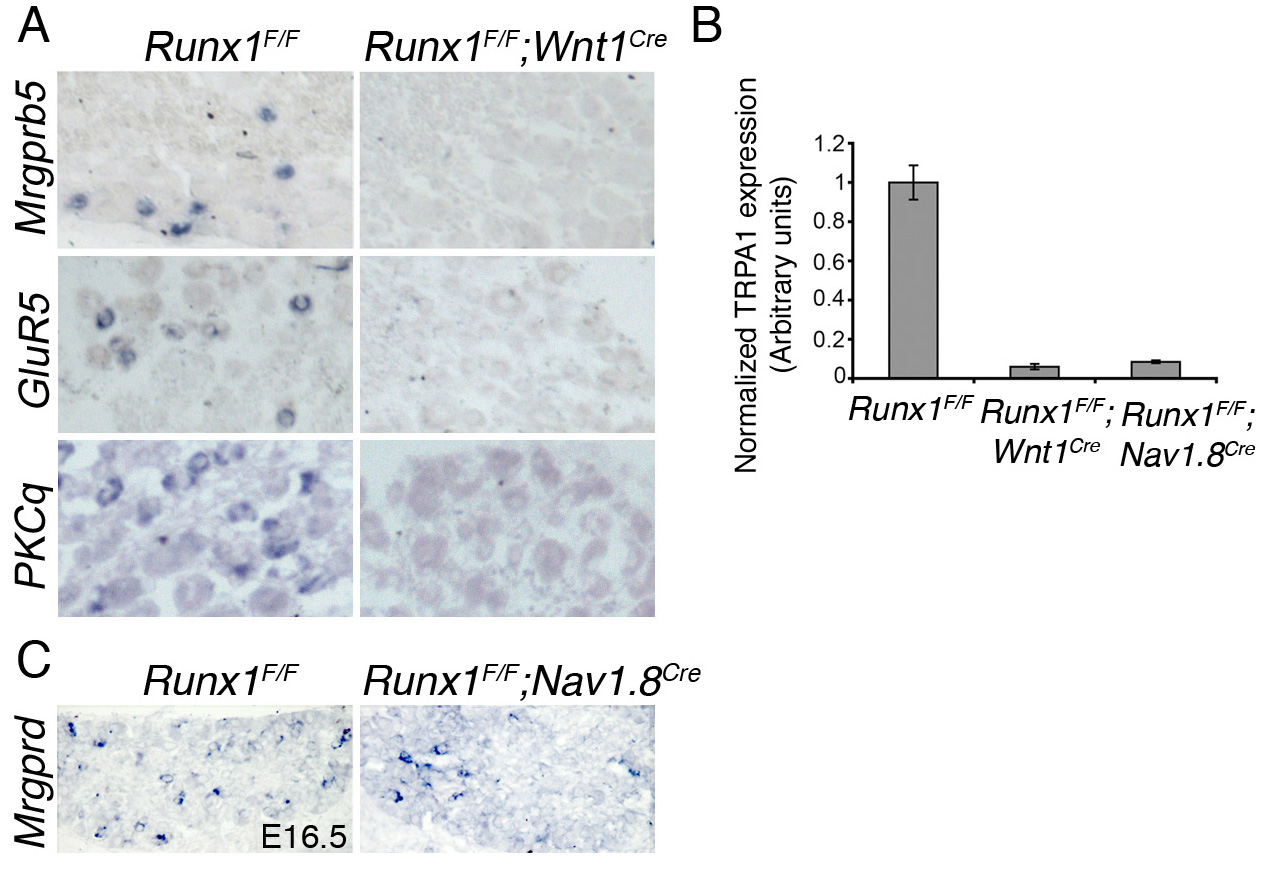

Supplement: Additional file 2 — Runx1 was required for Mrgprb5, GluR5, PKCq, and TRPA1 expression, and for the maintenance of Mrgprd expression. (A) In situ hybridization using the indicated probe on lumbar L4/5 DRG from P30 Runx1F/F control mice and Runx1F/F;WntCre early knockout mice. (B) Graph shows RT-PCR measuring mRNA levels of TRPA1 using adult L4-5 DRG from Runx1F/F control, Runx1F/F;Wnt1Cre early KO, and Runx1F/F;Nav1.8Cre late KO mice. RT-PCR data was normalized with HPRT. Each bar represent n = 2 mice. (C) In situ hybridization using an Mrgprd probe on sections through E16.5 lumbar DRG of Runx1F/F control and Runx1F/F;Nav1.8Cre mutant mice. Note that Mrgprd was detected in the mutant at E16.5 but lost at P30 (see Fig. 1), indicating a maintenance role. [file 1744-8069-6-45-S2.TIFF]

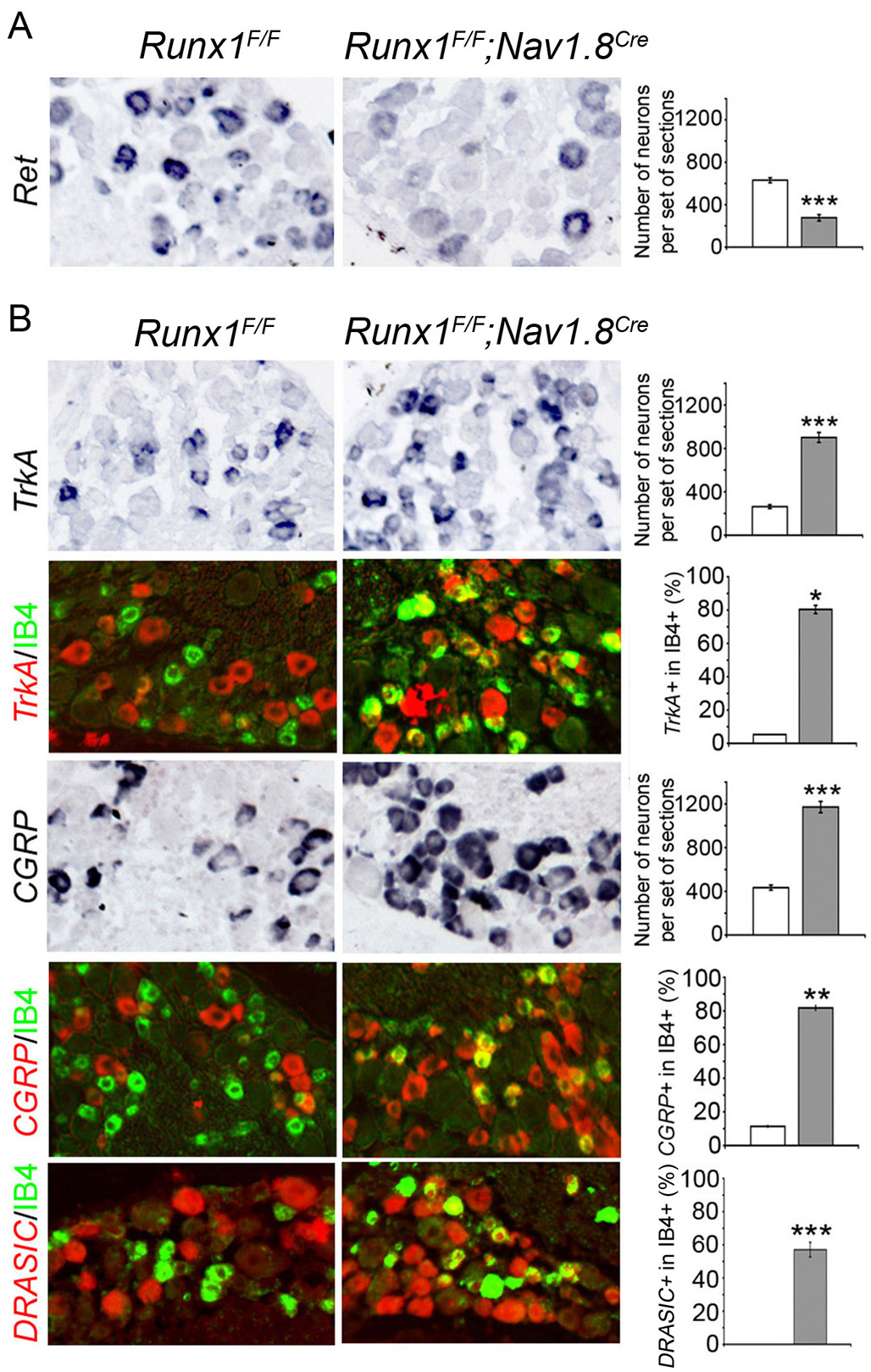

Supplement: Additional file 3 — Expression of Ret, TrkA, CGRP and DRASIC in Runx1F/F;Nav1.8Cre late knockout mice. (A) In situ hybridization with a Ret probe on transverse sections through adult lumbar DRG of control Runx1F/F mice and mutant Runx1F/F;Nav1.8Cre mice. To the right of the panels, a graph showed that the average (± SEM) number of neurons expressing Ret per set of lumbar DRG sections was reduced from 630 ± 24 (control, white bar) to 276 ± 30 (mutant, gray bar) (p < 0.001). (B) Expansion of peptidergic neuron markers in late Runx1 knockout. Single In situ hybridization with indicated probes (top) or double labeling of indicated mRNA (red) with IB4 (green) (bottom) in control and mutant DRG. The number of TrkA+ neurons increased from 262 ± 18 to 900 ± 46 (p < 0.001), and TrkA expression in IB4+ neurons increased from 5.30% ± 0.05% to 80.4% ± 2.4% (p < 0.05). CGRP+ neurons increased from 434 ± 23 to 1171 ± 52 (p < 0.001), and CGRP+ in IB4+ neurons increased from 11.4% ± 0.4% to 81.5% ± 1.3% (p < 0.01). DRASIC+ in IB4+ neurons increased from 0% to 57.1% ± 4.4% (p < 0.001). However, the expression of the vesicular Glutamate transporters (VGLUT1 and VGLUT2) and Nav1.8 were not affected in late Runx1 knockout mice (data not shown). [file 1744-8069-6-45-S3.TIFF]

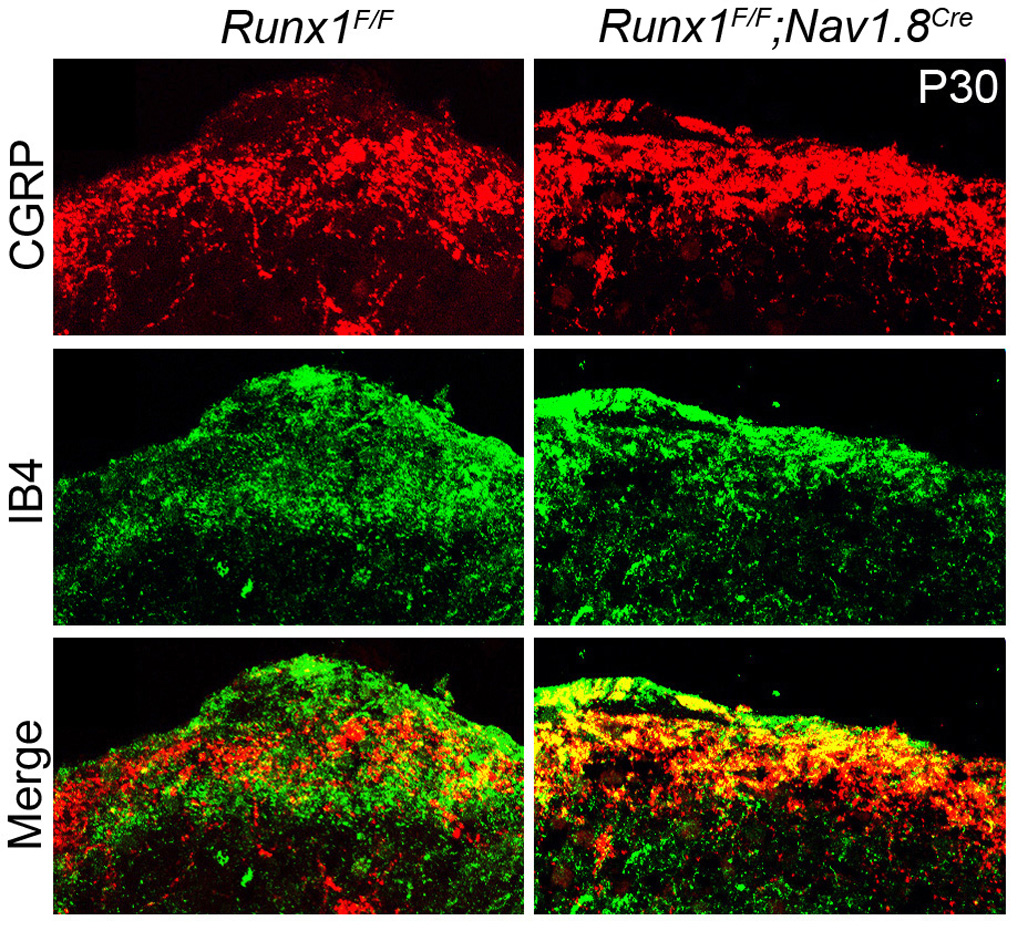

Supplement: Additional file 4 — Afferent central innervation of IB4+ sensory fibers in the dorsal horn was impaired in Runx1F/F;Nav1.8Cre late knockout. Double staining of IB4 (green) and CGRP (red) on P30 dorsal horn sections of control Runx1F/F and Runx1F/F;Nav1.8Cre mice. In control mice, peptidergic CGRP+ fibers innervated the superficial lamina, while nonpeptidergic IB4+ fibers innervated preferentially the inner lamina. Similar to the phenotype in Runx1 early conditional mutant, IB4+ fibers in Runx1F/F;Nav1.8Cre late mutants shifted their innervation from the inner lamina to the more superficial lamina. [file 1744-8069-6-45-S4.TIFF]

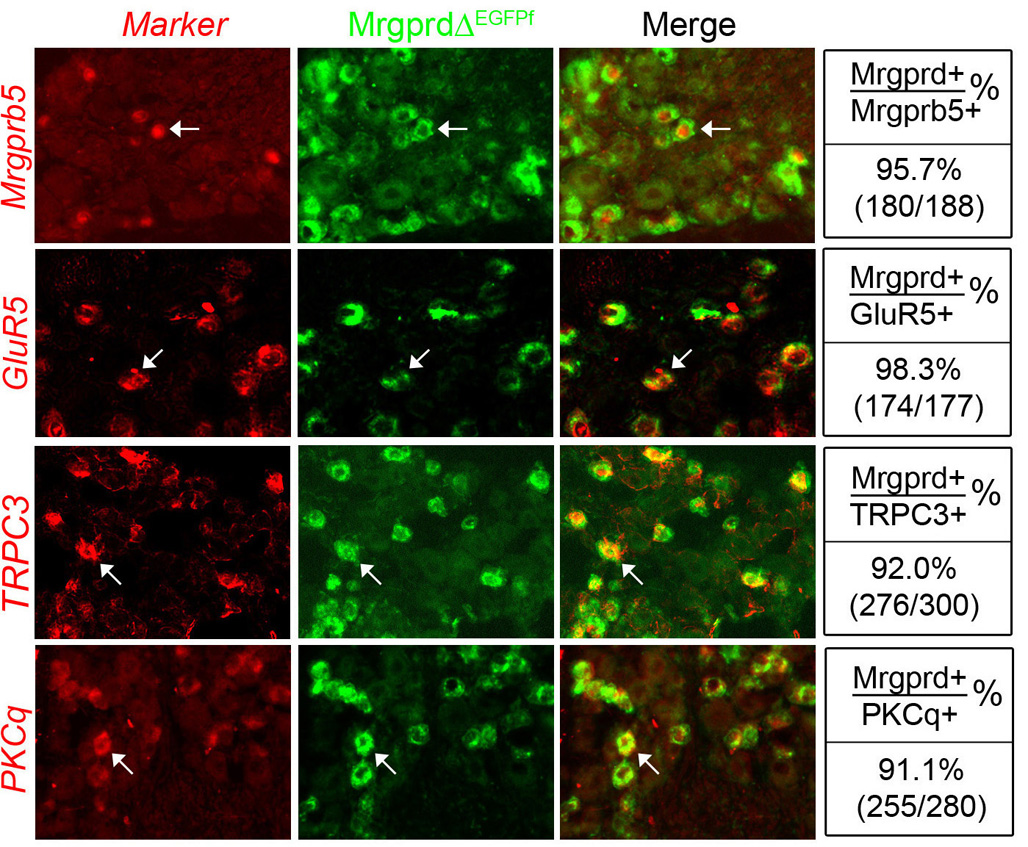

Supplement: Additional file 5 — Double labeling of GFP protein (green) and indicated RNA probe (red) in lumbar DRG from P30 MrgprdΔEGFP animals. Mrgprd was used here as a surrogate marker for Runx1+ neurons; see main text for details. Note that Mrgprb5, GluR5, TRPC3, and PKCq were largely overlapping with GFP and were thus predominantly in Runx1-persistent neurons. Quantitative data were shown to the right of the panels. [file 1744-8069-6-45-S5.TIFF]

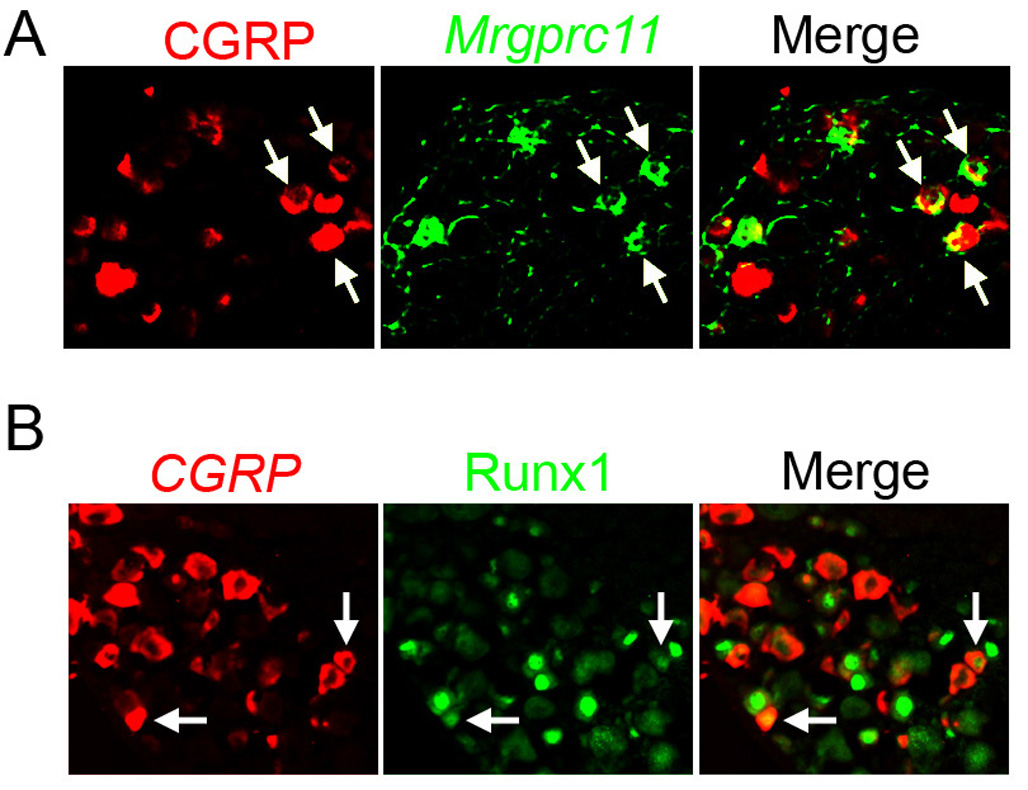

Supplement: Additional file 6 — Double Staining of CGRP with Mrgprc11 and with Runx1. (A) A majority of Mrgprc11+ neurons were peptidergic. Double staining of CGRP protein (red) and Mrgprc11 mRNA (green) on sections from P30 lumbar DRG from WT mice. Note that 71% of total Mrgprc11+ neurons (67 of 94) coexpressed CGRP (arrows). (B) Runx1 was expressed in a small subset of peptidergic neurons. Double labeling of Runx1 protein (green) and CGRP mRNA (red) on P30 lumbar sections of DRG from WT mice. About 15% of CGRP+ neurons (40 in 261) showed detectable Runx1 expression (arrows). [file 1744-8069-6-45-S6.TIFF]

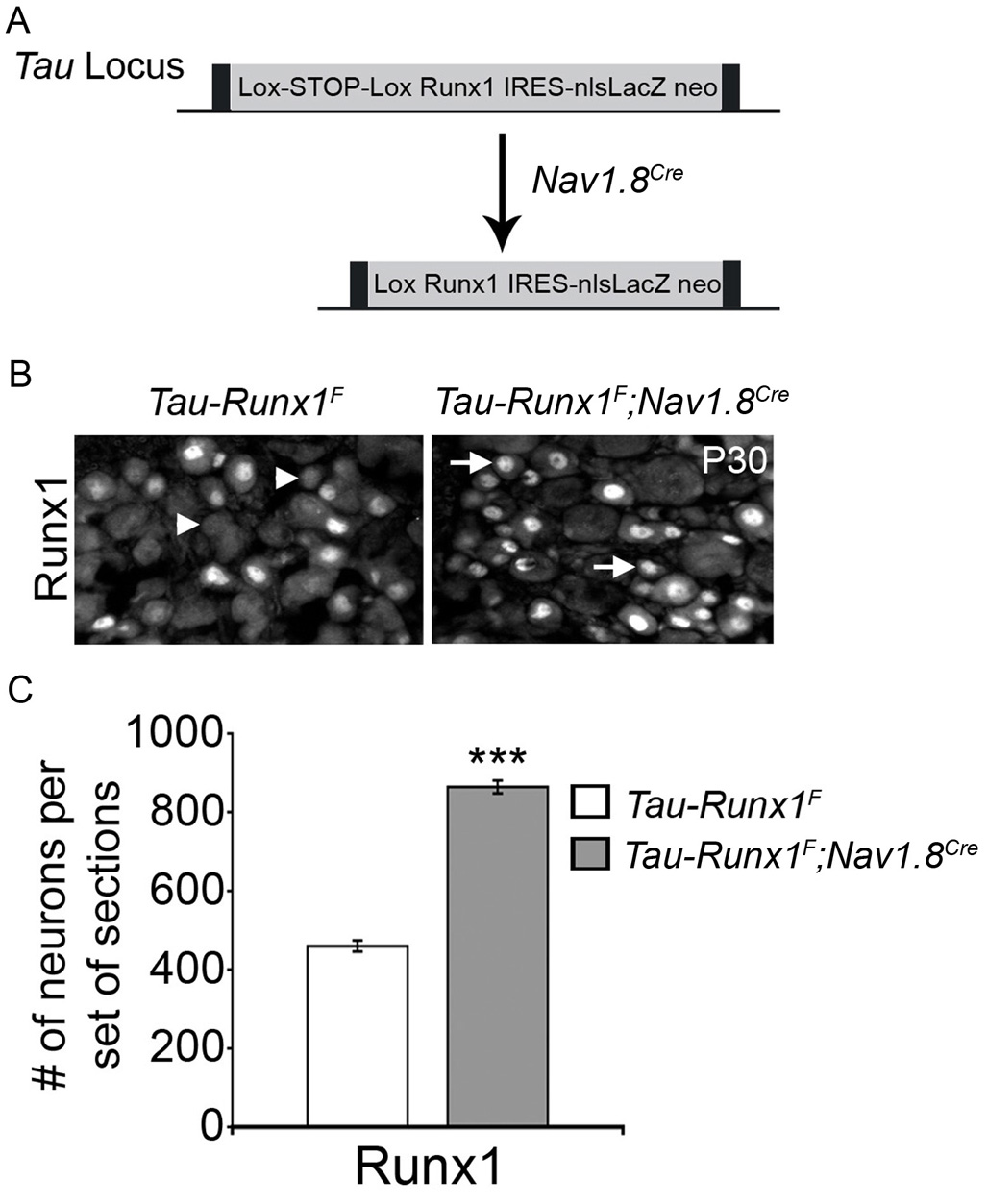

Supplement: Additional file 7 — Generation of Tau-Runx1F;Nav1.8Cre mice that drove constitutive Runx1 expression in most nociceptors. (A) Schematic showing the conditional knock-in of the Tau-Runx1 allele. A lox-STOP-lox-Runx1-IRES-nlsLacZ-neo cassette was inserted into exon 2 (black box) of the Tau locus. After crossing Tau-Runx1F mice with Nav1.8Cre mice, The 'STOP' was excised by Cre-mediated DNA recombination, allowing Runx1 to be expressed from the Tau locus. (B) Expansion of Runx1 expression in Tau-Runx1F;Nav1.8Cre mutant mice. Transverse sections through adult lumbar DRG of Tau-Runx1F control mice (left) and Tau-Runx1F;Nav1.8Cre mice (right) were labeled by immunostaining with Runx1. Arrowheads indicate Runx1-negative neurons while arrows indicate Runx1-positive neurons. (C) Graph showing that the average (± SEM) of total number of neurons expressing Runx1 (as detected by immunohistochemistry) per set of sections from control (white bar) and mutant mice (gray bar) increased from 460 ± 14 to 864 ± 17 (***p < 0.001). Futhermore, the total neuron number (as marked by the pan-neuronal marker SCG10) was reduced in the mutant mice by 25% (from 1427 ± 52.8 to 1068 ± 33, p < 0.01). [file 1744-8069-6-45-S7.TIFF]

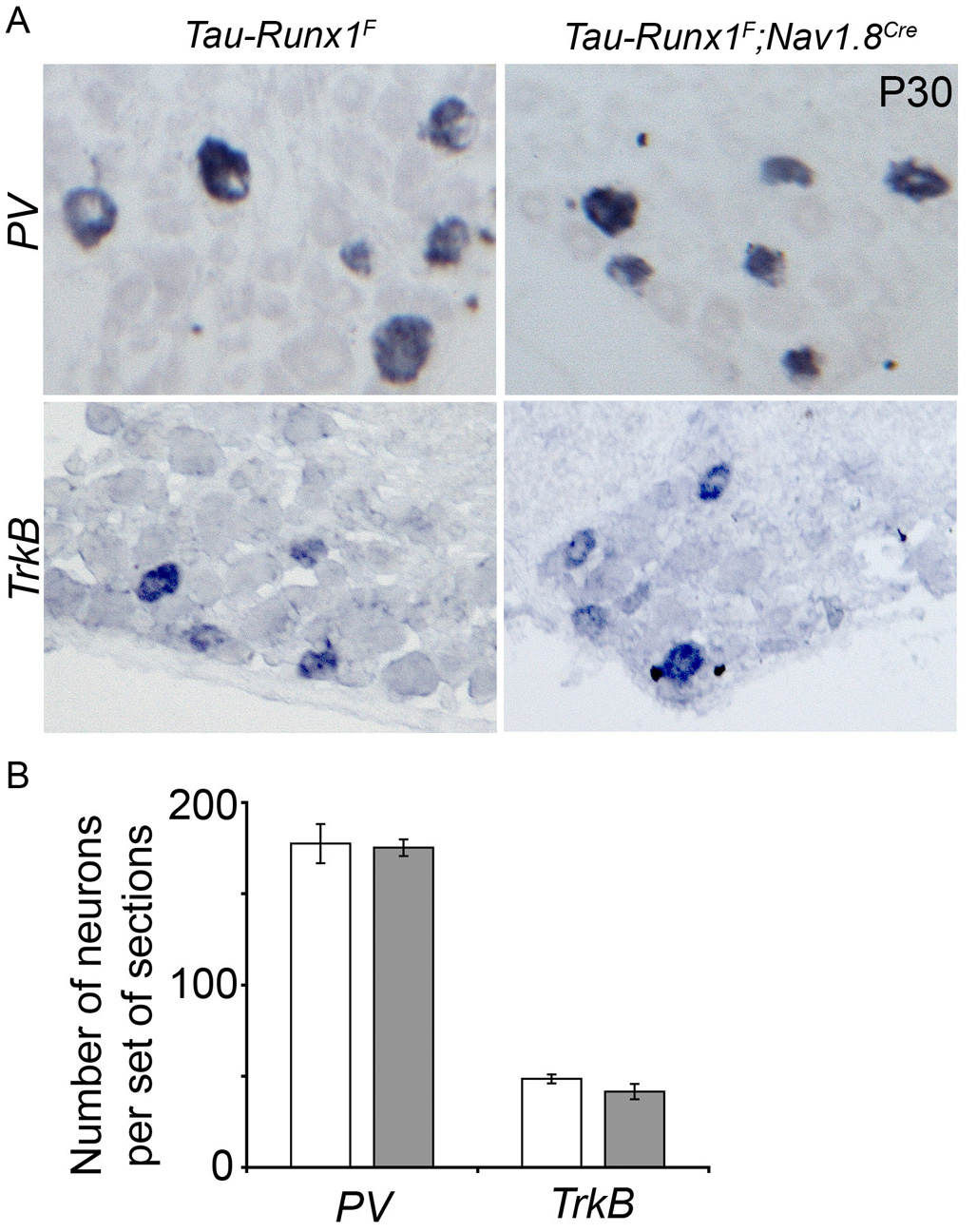

Supplement: Additional file 8 — Development of proprioceptors and mechanoceptors was unaffected in Tau-Runx1F;Nav1.8Cre mice. (A) In situ hybridization using the indicated probes on sections through adult lumbar (L4/L5) DRG of Tau-Runx1F control and Tau-Runx1F;Nav1.8Cre mutant mice. (B) Graph showing the average (± SEM) of the total number of neurons expressing the indicated probes per set of lumbar DRG sections of control (white bar) and mutant mice (gray bar). Note that the numbers of PV+ and TrkB+ neurons per set of sections were not significantly changed in mutant versus control animals (from 177 ± 11 to 175 ± 5 for PV+ neurons and from 48 ± 2 to 42 ± 4 for TrkB+ neurons) (p > 0.05). [file 1744-8069-6-45-S8.TIFF]

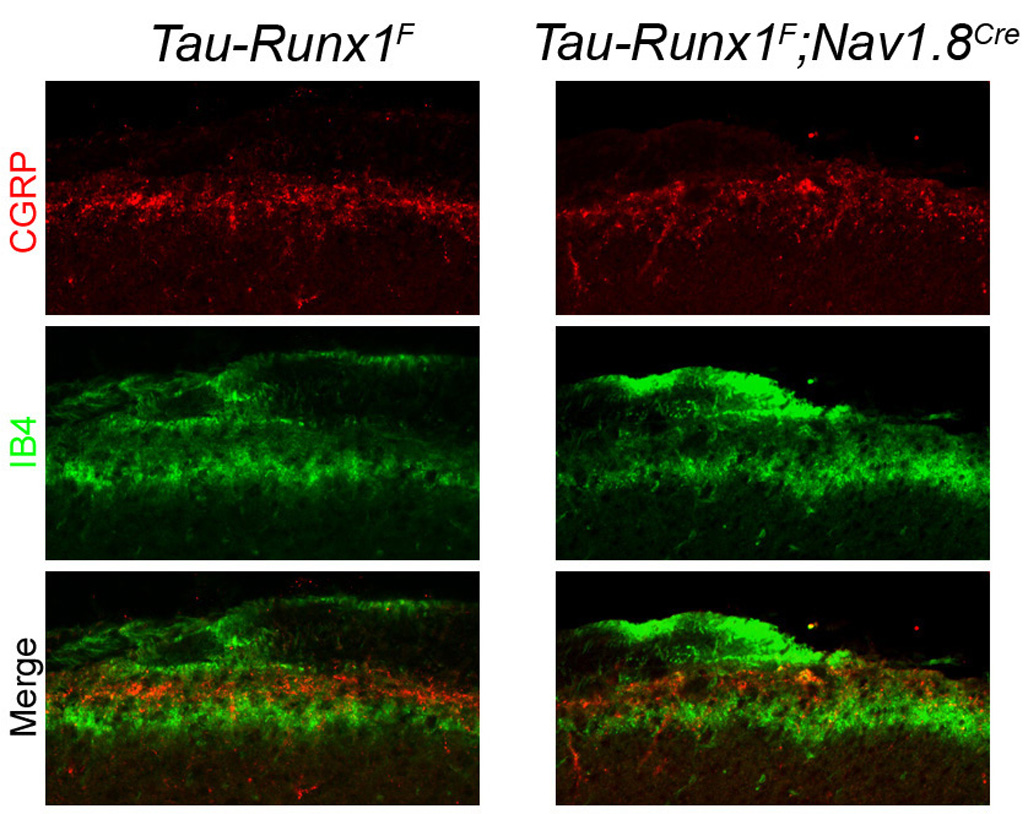

Supplement: Additional file 9 — Examination of afferent central innervation in the dorsal horn of Tau-Runx1F;Nav1.8Cre mutants. Double staining of IB4 (green) and CGRP (red) on P30 dorsal horn sections of Tau-Runx1F control and Tau-Runx1F;Nav1.8Cre mutant mice. Note that the residual CGRP+ fibers still innervated the superficial lamina, and IB4+ fibers also showed normal innervation to the inner lamina of the spinal cord of mutant mice. [file 1744-8069-6-45-S9.TIFF]
